# Detection of mitochondrial DNA with 4977 bp deletion in leukocytes of patients with ischemic stroke

**DOI:** 10.1371/journal.pone.0193175

**Published:** 2018-02-23

**Authors:** Yu-hua Huang, Chiung-Mei Chen, Yun-Shien Lee, Kuo-Hsuan Chang, Huei-Wen Chen, Yi-Chun Chen

**Affiliations:** 1 Department of Neurology, Chang Gung Memorial Hospital Linkou Medical Center, Taoyuan, Taiwan; 2 College of Medicine, Chang-Gung University, Taoyuan, Taiwan; 3 Department of Biotechnology, Ming Chuan University, Taoyuan, Taiwan; 4 Genomic Medicine Research Core Laboratory, Chang Gung Memorial Hospital, Taoyuan, Taiwan; Fraunhofer Research Institution of Marine Biotechnology, GERMANY

## Abstract

**Background:**

Coronary artery disease is associated with a common mitochondrial DNA alteration, a 4977 bp deletion (mtDNA^4977^). The role of mtDNA^4977^ in ischemic stroke is unknown.

**Methods:**

Real-time quantitative PCR was performed to quantify total mtDNA and mtDNA^4977^ in leukocytes in 283 ischemic stroke cases and 135 controls. Ratios of mtDNA^4977^ to total-mtDNA and total-mtDNA to nuclear-DNA were calculated. Nested PCR and Sanger sequencing were used to confirm undetectable levels of mtDNA^4977^.

**Results:**

For 191 patients and 74 control subjects in the male group and 92 patients and 61 control subjects in the female group, there were no significant between-group differences in age, cholesterol level, body mass index, stroke severity, or 4977 deletion. After adjusting for confounding factors, there was no correlation between mtDNA^4977^ amount and infarction risk, recurrent stroke, or stroke severity. However, mtDNA^4977^ was undetected in 6.94% subjects, and these individuals had a higher prevalence of stroke than those with detectable mtDNA^4977^ (OR: 0.181, 95% CI 0.041–0.798, p = 0.024). Additionally, mtDNA^4977^ status had no effect on stroke prognosis, including stroke severity and recurrent stroke.

**Conclusion:**

In conclusion, there was no apparent association between mtDNA^4977^ deletion and cerebral infarction. Undetectable mtDNA^4977^ may be a marker or risk factor for ischemic stroke.

## Introduction

Mounting evidence suggests that mitochondria dysfunction and the accumulation of mitochondria DNA (mtDNA) damage plays an important role in the development of atherosclerotic lesions. MtDNA damage not only correlates with the severity of atherosclerotic lesions but also precedes initial pathological processes [1–5]. The exact mechanism of this relationship is not well understood, but may be related to the central role of mitochondria in cellular energy homeostasis [5].

Evidence also suggests that somatic mtDNA alternations are associated with coronary artery disease (CAD) and atherosclerosis. Ballinger et al. [6] showed that atherosclerotic aortas had increased numbers of mtDNA oxidative lesions compared to normal aortas. Previous studies have shown that patients with CAD have an increased abundance of mtDNA 4977 bp deletions (mtDNA^4977^) in the heart and in circulating leukocytes [7, 8]. A significantly higher prevalence of mtDNA^4977^ and higher relative amounts of the deletion were identified in CAD patients compared to healthy control subjects (26.2% versus 4.5%; p = 0.03 and 0.089 ± 0.02% versus 0.009 ± 0.009; p = 0.02) [9]. The accumulation of mtDNA^4977^ has also been implicated in human aging [10]; in one study, the amount of mtDNA^4977^ was positively associated with age, independent of traditional risk factors and clinical parameters [11].

Stroke is the third leading cause of death worldwide and is associated with a 30-day disability rate of 61.2% in Taiwan [12]. Atherothrombotic stroke, which is diagnosed when an ischemic lesion > 1.5 cm is identified on brain imaging and when there is arterial stenosis > 50% of any carotid/cerebral artery, accounts for 20% of all stroke events [13]. Atherothrombotic stroke and CAD share the same conventional risk factors, susceptibility genes, and pathophysiology [13]. Additionally, several major risk factors for ischemic stroke are associated with mitochondrial dysfunction such as that occurring in metabolic syndrome, cigarette smoke exposure, and hypercholesterolemia [14–16].

Based on the earlier work mentioned in the Introduction section above, we hypothesized that, similar to coronary artery disease, stroke is associated with a mtDNA^4977^ deletion.

Recently, a genome-wide study reported an association between mitochondrial respiratory chain complex I/IV dysfunction and ischemic stroke, especially for small vessel stroke [2]. The common deletion mtDNA^4977^ occurred between nucleotides 8470 and 13459, in which five genes for tRNA and seven genes for components of complex I (ND3, ND4, ND4L, and a part of NAD5), IV (COXIII), and V of the respiratory chain (ATPase 6 and a part of ATPase 8) were compromised [4]. Mitochondrial dysfunction, particularly involving complexes I and IV, has been associated with cardiovascular disease [4], stroke [3, 8], Alzheimer’s disease [17], Parkinson’s disease [17–20], and psychiatric disorders [21]. While it is controversial how functions are altered by mtDNA^4977^ deletion, electron movement in the respiratory chain of mtDNA^4977^ cells is still likely to lead to reactive oxygen species (ROS) generation. Damaged mtDNA leads to the dysfunction of integral membrane protein complexes of the respiratory chain, which consequently leads to apoptotic death and cellular necrosis [3–5, 15]. Therefore, we hypothesized that the pathology of mitochondrial damage in atherogenesis may be related to alterations in the generation of reactive oxygen species and in ATP synthesis.

In the present study, we thus hypothesized that mtDNA^4977^ might play an important role in ischemic stroke. To date, little research has examined the presence of mtDNA^4977^ deletions in the cells and tissues of patients with stroke. Therefore, we evaluated mtDNA^4977^ in a cohort of patients with stroke and examined relevant associations.

## Materials and methods

### Patients and control subjects

Study subjects were enrolled from Chang Gung Memorial Hospital. Each patient or her/his legally acceptable representative was informed of the aim of the study. This study has been cleared by Chang Gung Memorial Hospital Institution Ethics Review Board for human studies and patients or guardians provided written informed consent prior to study participation. Ischemic stroke and its subtypes were diagnosed and classified by two neurologists based on clinical presentation and the available brain imaging data. This study enrolled patients with atherothrombotic infarction or lacunar infarction. Subtypes of cerebral infarction were defined by the TOAST criteria with modifications as follows: (1) atherothrombotic infarction was diagnosed when there was an ischemic lesion > 1.5 cm on brain imaging and arterial stenosis > 50% of any carotid/cerebral artery; (2) lacunar infarction was diagnosed when the brainstem or subcortical hemispheric ischemia was < 1.5 cm in diameter on brain imaging, and when there was no clinical evidence of cerebral cortical or cerebellar dysfunction [22]. Patients with recent stroke (< 6 months) or a medical condition such as atrial fibrillation, acute coronary syndrome, chronic renal disease, infection or inflammatory disease, other neurodegenerative disease, or cardioembolism/undetermined stroke type were excluded. Healthy control subjects were recruited randomly from the community and had no history of stroke (including hemorrhagic or ischemic stroke), neurological disease, or overt medical disease such as chronic renal failure, myocardial infarction, atrial fibrillation, and cancer.

### Evaluation of mtDNA^4977^

**Fig 1:**
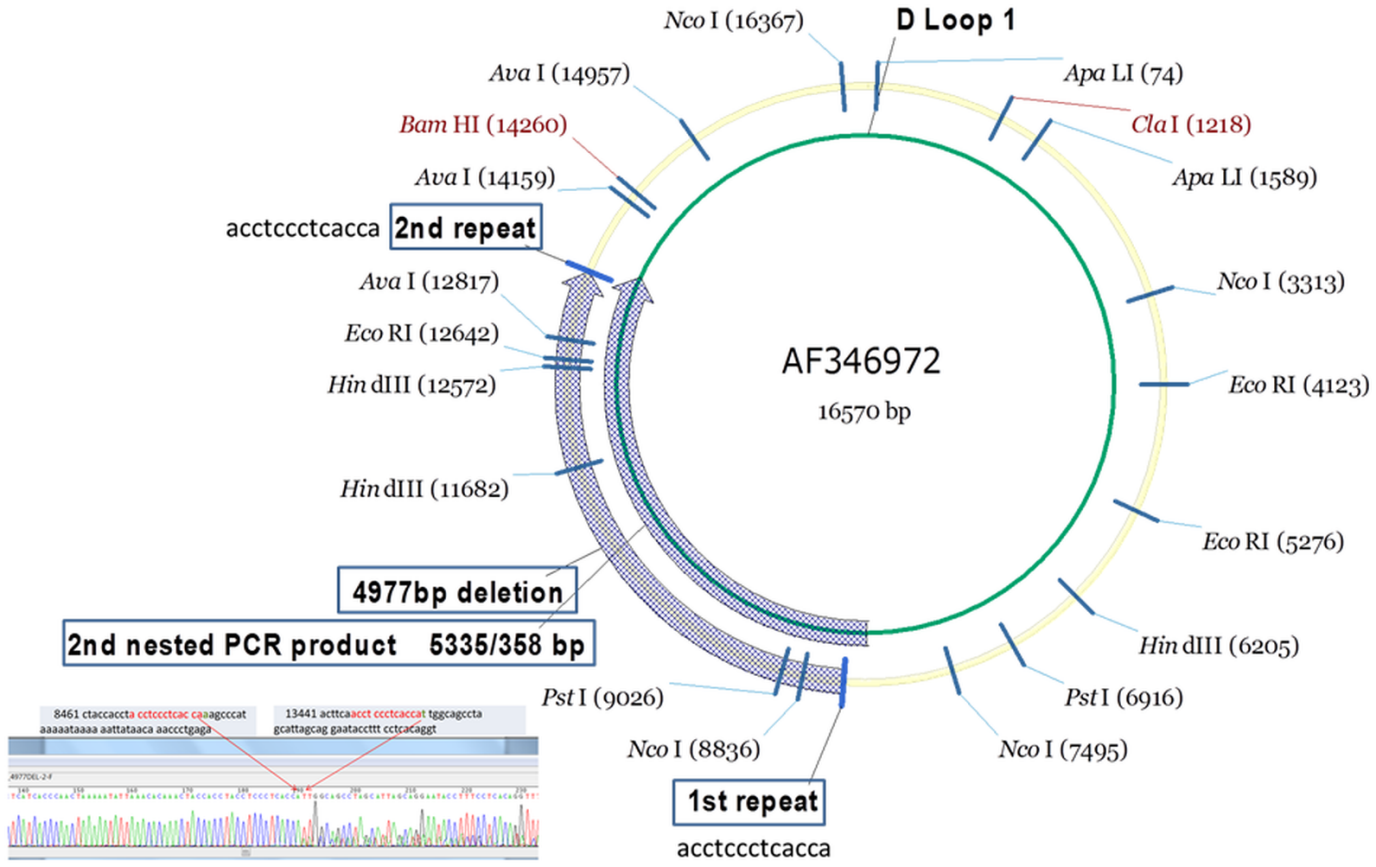
Detection of mtDNA^4977^ and quantification of total and deleted mtDNA in leukocytes using real-time quantitative PCR.

Blood samples were collected and immediately (within 2 hours) processed for leukocyte isolation. Samples were then frozen at -80°C and stored until analysis. Leukocyte DNA was extracted using a DNA Extraction Kit according to the manufacturer’s specifications (Agilent Technologies, La Jolla, CA). Nested PCR was performed to detect the presence of mtDNA^4977^ [23]. The two pairs of nested primers used for the detection of mtDNA^4977^ are shown in Fig 1. The PCR condition was set as Table 1. The PCR condition was set as pre-denaturation at 94°C for 5 min, 30 cycles at 94°C for 10 secs, 58°C for 45 secs and 72°C for 50 secs, and a final extension at 72°C for 10 min. We used the ratio of the absorbance at 260 and 280 nm (A260/280) to assess the purity of nucleic acids. The DNA samples were all with good purity (260/280 = 1.7–2.0). Then we run the DNA samples by standard protocol for performing 1% agarose gel electrophoresis to detect other possible contaminants free RNA or faint smear. The samples were run in triplicates. Products were sequenced by Sanger sequencing. The presence of mtDNA^4977^ was indicated by the appearance of a 358-bp band and verified by sequencing analysis.

**Table 1:** Primers using sequencing for nested PCR.

Primer	sequence (5'—>3')	Tm(°C)	PCR product size(bp)
4977del-1-F	AACCACAGTTTCATGCCCATC	62.1	5472/495
4977del-1-R	TGTTAGTAAGGGTGGGGAAGC	60.4	5472/495
4977del-2-F	ACCCTATAGCACCCCCTCTAC	58.5	5335/358
4977del-2-R	CTTGTCAGGGAGGTAGCGATG	62.1	5335/358

### Quantification of mtDNA^4977^

To quantify the relative ratio of mtDNA^4977^ to total mitochondria DNA, real-time quantitative PCR (RTQ-PCR) was performed to quantify total mtDNA and mtDNA^4977^ molecules in leukocytes. TaqMan^®^ Gene Expression Assays and the Sequence Detection system (ABI Prism 7900, Applied Biosystems) were used for quantification (Table 2). β-actin gene expression was measured as an indicator of nuclear DNA concentration. The common mtDNA deletion of 4,977 bp had breaking points between nucleotides 8470 and 13347, with two direct 13-bp repeats. The ND1 gene used for reference was located at nucleotides 3312 to 4122 on mtDNA and provides one of the seven mitochondrial encoded subunits of NADH dehydrogenase. Primers and TaqMan probes ordered with Applied Biosystems^™^ FAM ^™^ and VIC ^™^ dyes, used to amplify mtDNA^4977^, ND1 gene and β-actin gene. The Ct method (ΔCt) was used to calculate relative levels of mtDNA^4977^ between patients and control subjects. First, we normalized the Ct endogenous control: ΔCt sample = Ct Target (4977 deletion)—Ct Endogenous Control (ND1), ΔCt sample indicated the log2 ratio of mtDNA^4977^ vs mtDNA; and ΔCt reference = Ct Target (modified β-actin)—Ct Endogenous Control (ND1 for β-actin), ΔCt reference indiacted the log2 ratio of genomic DNA vs mtDNA. ΔCt sample and ΔCt reference values were used to evaluate mtDNA^4977^ deletion levels.

**Table 2:** Primers and TaqMan probes for real-time quantitative PCR.

	Assay ID	Primers and VIC-probe
		Forward Primer:
		5'- GCCCACCATAATTACCCCCATAC-3'
*4977BP-DELETED*	AIS09GS	Reverse Primer:
		5'-GAGTAGAAACCTGTGAGGAAAGGT-3'
		Probe: FAM-CCTCATCACCCAACTAAAA-NFQ
		(Product size: 5117/140 bp)
*MT-ND1*	Hs02596873_s1	VIC
*β-actin*	Hs03023880_g1	FAM

Annealing temperature: 60°C.

The common mtDNA deletion of 4,977 bp had breaking points between nucleotides 8461 and 13347, with two direct 13-bp repeats. The fragment was the amplification target between 8461(1^st^ repeat:acctccctcacca) and 13347 bp (2^nd^ repeat:acctaaatcacca). The grey arrow at the outer circle shows mtDNA deletion of 4,977 bp, and the grey arrow at the inner circle demonstrates the product of the second nested PCR. In the presence of deletion, the amplification product is 358 bp in size, while the wild-type mtDNA yields a product of 5335 bp, which shall not be shown on sequencing. The left corner shows the Sanger sequence that proved the 2^nd^ nested PCR products and confirmed of the 4,977 bp deletions.

### Statistical analysis

Pearson’s χ^2^-tests or t-tests were utilized to compare demographic data between the patient and control groups; all significance tests were two-tailed. Association analyses were performed first stratified by sex and then combined. Co-variables included age, sex, hypertension, diabetes mellitus (DM), total cholesterol (TC) level, smoking, and alcohol use. A logistic regression was performed to assess the relationship between mtDNA^4977^ and stroke outcome. All data analyses were performed using IBM SPSS Statistics software version 20. The threshold for statistical significance was p < 0.05.

## Results

Study group characteristics are presented in Table 3. A total of 283 patients and 135 control subjects were included. In the male group, hypertension and smoking were significantly more common in patients with stroke than in control subjects (p = 0.008 and p = 0.016, respectively). In the female group, hypertension and DM were more common in patients with stroke than in control subjects (p = 0.006 and p < 0.001, respectively). When the groups were combined, patients with ischemic stroke showed higher prevalence of hypertension (p < 0.001), DM (p < 0.001), smoking (p < 0.001), and alcohol consumption (p = 0.046) compared to the control group. There were no significant differences in age, cholesterol level, body mass index, initial Glasgow coma scale (GCS) scores (≤ 9), ΔCt samples, and ΔCt reference values between the sexes. GCS scores were later dichotomized at 8, as this is the clinical cutoff point often used for coma [24]. We thus used an initial GCS score of ≤ 9 as an indicator of initial conscious level and stroke severity. Additionally, higher percentages of mtDNA^4977^ were detected in the control group than in the ischemic stroke group (male group: control vs. stroke, 97.3% vs. 91.1%; female group: control vs. stroke, 100% vs. 89.1%; ischemic stroke group vs. control group, p = 0.002). The average intra -coefficient of variation (CV) and inter-CV of ΔCt (mtDNA^4977^ -ND1) is 2.1% and 6.8%, respectively.

**Table 3:** Demographic data in patients with ischemic stroke and control subjects.

Characteristic	Male group (n = 265)			Female group (n = 153)			p-value for infarct cases vs. control subjects
	Infarct	Control	p-value	Infarct	Control	p-value	
Number	191	74	-	92	61	-	-
Mean age (years)	66.43 ± 12.16	64.95 ± 12.79	0.628	68.40 ± 11.82	66.57 ± 12.56	0.362	0.287
Hypertension (n/%)	131 (68%)	42 (56%)	0.008	70 (76%)	34 (55%)	0.006	< 0.001
Diabetes mellitus (n/%)	63 (32%)	17 (22%)	0.068	44 (47%)	6 (9.8%)	< 0.001	< 0.001
Alcohol use (n/%)	51 (26%)	14 (18%)	0.195	0 (0%)	0 (0%)	-	0.046
Smoking (n/%)	101 (52%)	27 (36%)	0.016	3 (3.2%)	0 (0%)	0.278	< 0.001
Mean BMI (kg/m^2^)	25.46 ± 3.55	25.61 ± 3.15	0.779	24.81 ± 3.88	24.63 ± 5.39	0.812	0.745
TC (mg/dl)	178.2 ± 34.40	177.7 ± 34.17	0.873	193.2 ± 42.20	202.4 ± 37.55	0.179	0.149
Initial GCS score ≤ 9	6.1%	-	-	14.7%	-	-	0.375^c^
30-day MRS score ≥ 3	40%	-	-	50%	-	-	0.042 ^c^
4977 deletion (+)	174 (91.1%)	72 (97.3%)	0.079	82 (89.1%)	61 (100%)	0.008	0.002
ΔCt^a^	2.24 ± 0.86	2.24 ± 0.84	0.998	2.22 ± 0.77	1.94 ± 0.92	0.049	0.155
ΔCt(4977-ND1)^b^	4.74 ± 2.50	4.92 ± 1.91	0.571	5.07 ± 2.30	5.31 ± 2.04	0.508	0.292

^a^ΔCt = Ct Target (β-actin)–Ct Endogenous Control (ND1 for β-actin)

^b^ΔCt (4977-ND1) = Ct Target (4977 deletion)–Ct Endogenous Control (ND1)

^c^p-value for males vs. females in the infarct group

Data are expressed as numbers or mean ± standard error. Comparisons between control subjects and infarction cases were analyzed using χ^2^-tests or t-tests where appropriate. BMI, body-mass index; GCS, Glasgow coma scale; MRS, modified Rankin scale; TC, total cholesterol.

Of the study cohort subjects (29 study subjects), 6.94% did not carry mtDNA^4977^, including 27 patients with infarction and two healthy control subjects. In these patients, deletion of 4977 was undetectable using TaqMan quantification and confirmed by Sanger sequencing. There was no difference in mean age, stroke risk factors, or ΔCt reference values between participants with and without detectable mtDNA^4977^ (Table 4). Participants without detectable mtDNA^4977^ had a higher rate of infarction than patients with detectable mtDNA^4977^ (male group: 89.5% vs. 70.7%, p = 0.079; female group: 100% vs. 57.3%, p = 0.008; ischemic stroke group vs. control group, p = 0.002) (Table 4).

**Table 4:** Comparison of patients with and without the 4977 deletion.

Characteristic	Male group (n = 265)			Female group (n = 153)			p-value for subjects with vs. without the 4977 deletion
	4977 deletion	no 4977 deletion	p-value	4977 deletion	no 4977 deletion	p-value	
No. of Infarct/Patien	174/246, 70.7%	7/19, 89.5%	0.079	82/143, 57.3%	10/10, 100%	0.008	0.002
Mean age (years)	66.50 ± 12.30	72.74 ± 0.96	0.166	67.90 ± 12.16	64.40 ± 11.97	0.392	0.135
Hypertension (n/%)	173 (70.3%)	15 (78.9%)	0.425	95 (66.4%)	9 (90%)	0.129	0.123
Diabetes mellitus (n/%)	80 (32.5%)	6 (31.6%)	0.904	47 (32.9%)	3 (30%	0.840	0.836
Alcohol use (n/%)	65 (26.4%)	8 (42.1%)	0.130	0 (0%)	0 (0%)	-	0.125
Smoking (n/%)	128 (52.0%)	10 (52.6%)	0.914	3 (2.01%)	0 (0%)	0.826	0.847
Mean BMI (kg/m^2^)	25.51 ± 3.44	25.18 ± 2.45	0.615	24.84 ± 4.58	22.83 ± 2.91	0.102	0.156
TC (mg/dl)	178.1 ± 34.26	182.7 ± 40.71	0.641	197.7 ± 40.36	184.8 ± 36.63	0.310	0.833
Initial GCS score ≤ 9	6.1%	14%	0.423	14.7%	0	0.351	0.375
30-day MRS score ≥ 3	40%	22%	0.254	50%	100%	0.021	0.462
ΔCt ^a^	−2.26 ± 0.86	−1.99 ± 6.80	0.166	−2.12 ± 0.86	−1.93 ± 0.64	0.377	0.098

^a^ΔCt = Ct Target (β-actin)–Ct Endogenous Control (ND1 for β-actin)

Data are expressed as the number or mean ± standard error. Comparisons between subjects with and without the 4977 deletion were analyzed using χ^2^-tests or t-tests where appropriate. BMI, body-mass index; GCS, Glasgow coma scale; MRS, modified Rankin scale; TC, total cholesterol.

After adjusting for confounding factors, there was no correlation between mtDNA^4977^ amount and infarction risk (odds ratio [OR] = 0.983, 95% confidence interval [CI] = 0.881–1.097, p = 0.762), recurrent stroke (OR = 1.106, 95% CI = 0.952–1.285, p = 0.187), and stroke severity (OR = 0.953, 95% CI = 0.778–1.168, p = 0.645) (Table 5). Consistent with our initial analysis, individuals who did not carry mtDNA^4977^ were more likely to have a stroke than individuals with detectable mtDNA^4977^ (OR = 0.181, 95% CI = 0.041–0.798, p = 0.024). Additionally, the presence or absence of mtDNA^4977^ had no effect on stroke prognosis (recurrent stroke: OR = 1.109, 95% CI = 0.279–4.411, p = 0.883; stroke severity: OR = 0.619, 95% CI = 0.195–1.969, p = 0.417).

**Table 5:** Logistic regression models of mt4977 deletion and stroke prognosis.

Characteristic	Infarction		MRS ≥ 3		Recurrent infarcts	
	OR (95% CI)	p-value	OR (95% CI)	p-value	OR (95% CI)	p-value
ΔCt (4977-ND1)^a^	0.983 (0.881–1.097)	0.762	1.106 (0.952–1.285)	0.187	0.953 (0.778–1.168)	0.645
Presence of 4977^b^	0.181 (0.041–0.798)	0.024	0.619 (0.195–1.969)	0.417	1.109 (0.279–4.411)	0.883

^a^Logistic regression model for ΔCt (4977-ND1) and stroke prognosis, adjusting for age, sex, TC, diabetes mellitus, hypertension, and smoking.

^b^Logistic regression model for presence of the 4977 deletion and stroke prognosis, adjusting for age, sex, TC, diabetes mellitus, hypertension,and smoking. CI, confidence interval; MRS, modified Rankin scale; OR, odds ratio; TC, total cholesterol

## Discussion

The mtDNA^4977^ deletion is particularly relevant to the pathogenesis of atherosclerosis and has previously been reported as a potential predictor of coronary artery disease and stroke; yet, the relationship between mtDNA^4977^ and ischemic stroke remains unclear. This study found no correlation between mtDNA^4977^ amount and infarction risk, recurrent stroke, or stroke severity in a cohort of patients with acute ischemic stroke. Additionally, the presence or absence of mtDNA^4977^ had no effect on stroke prognosis, including recurrent stroke and stroke severity. In our cohort of 283 patients and 135 control subjects, 6.94% of cohort subjects did not carry mtDNA^4977^, and there was a significant higher prevalence of stroke in these individuals compared to those with detectable mtDNA^4977^. In conclusion, there was no apparent association between mtDNA^4977^ deletion and cerebral infarction. Undetectable mtDNA^4977^ may be a marker or risk factor for ischemic stroke.

MtDNA^4977^ are related to ROS that arise as a by-product of oxidative phosphorylation in mitochondria [25]. MtDNA^4977^ occur frequently in tissues of high oxygen demand and low mitotic activity, e.g. neuron cell or myocytes [26]. However, mtDNA^4977^ can still be detected in fast replicating cells, such as blood leukocytes, in much lower amounts [25, 26]. Botto et al. reported that mtDNA^4977^ can be found in both samples of blood cells and atherosclerotic lesions from patients with CAD [9]. Although mtDNA^4977^ in blood could not represent specifically for cerebral infarction, it is still a biomarker for systemic burden of oxidative stress. Because the brain tissue samples from stroke patients are difficult to access, biomarkers in blood is relatively more applicable.

MtDNA is more susceptible to oxidative damage than nuclear DNA [6] because of a limited mitochondrial capacity for DNA repair and the absence of histones for protection [8]. Numerous studies have implicated mtDNA^4977^ in various forms of carcinogenesis [27] as well as in aging [28]. mtDNA^4977^ is more frequent in the general population than previously thought; it was recently demonstrated that mtDNA^4977^ was present in 98.3% of subjects in a cohort of unrelated Chinese participants aged 5 days to 91 years, with an exponential increase in the frequency of mtDNA^4977^ with age [18]. This is consistent with the observation of mtDNA^4977^ in 93.06% of subjects (389/418) in our study cohort. The discrepancy between earlier and these current findings are likely related to cohorts of a wider age range as well as technological improvements in DNA detection.

MtDNA ^4977^ in cardiac myocytes and smooth muscle cells in atherosclerotic lesions are important contributors to pathogenesis of coronary artery disease (CAD) [29, 30]. Accumulation of mtDNA^4977^ is particularly found in high oxygen demand and low mitotic activity tissues. Regional accumulation of mtDNA^4977^ was higher in the left heart than in the right because the left heart has a higher workload and thus requires higher energy demand [31]. Regarding the hypothetical relationship between mitochondrial damage in atherogenesis and the generation of ROS and ATP synthesis, our data demonstrates that the presence of mtDNA^4977^ did not vary in accordance with a history of ischemic stroke. Additionally, mtDNA^4977^ levels were not significantly associated with recurrent stroke or stroke prognosis. Our results do not support the notion that mtDNA^4977^ plays a major role in cerebral vascular arthrosclerosis and cerebral infarction. In one study, mtDNA dysfunction and damage preceded early atherosclerotic changes, while oxidative markers remained normal, suggesting that changes were probably independent of oxidative stress [5]. Moreover, stroke syndromes caused by defects of mtDNA and nuclear DNA may be different, and recent clinical reports suggest that small vessel diseases are associated with diverse nuclear genetic variants [2, 32].

Because accumulation of mtDNA^4977^ is found in high oxygen demand and low mitotic activity tissues, such as neuron cells and myocytes, prior CAD models demonstrated increasing mtDNA^4977^and ROS production in coronary artery, aorta, and cardiac myocytes. In contrast, leukocytes are fast replicating with high mitotic activity, which may cause insufficient time for mtDNA^4977^ accumulation. Utilizing leukocytes from peripheral blood, our study showed that undetectable mtDNA^4977^ in leukocytes may be a biomarker for ischemic stroke. Because the brain tissue samples from stroke patients are difficult to access to confirm the link between undetectable mtDNA^4977^ and stroke, further animal model for confirmation is required before utilizing peripheral mtDNA^4977^ as a biomarker for ischemic stroke.

A meta-analysis of 38 studies found that proportions of mtDNA^4977^ were significantly decreased in cancerous tissue compared to adjacent non-cancerous tissue [33]. Another previous study showed that the frequency of mtDNA^4977^ was significantly higher in normal tissue compared to paired cancerous tissues including breast [34, 35], lung [36], esophageal squamous cell carcinoma, gastric [37], and colorectal cancers [23]. The possible mechanism underlying this relationship may be that mtDNA^4977^ serves a protective function against the tumor-promoting effects of other somatic mutations [38] or clonal expansion dilution of mtDNA^4977^ in tumor tissue during cancer progression. Moreover, mtDNA^4977^ might confer a metabolic disadvantage to hyper-proliferating cells and thus eliminate these cells by leading to apoptosis [29]. This may explain our finding that only a low percentage of individuals in our cohort (6.94%) did not carry mtDNA^4977^, and the finding that stroke was more prevalent in these individuals.

A limitation of the present study is that patients with cardiovascular problems such as atrial fibrillation and acute coronary syndrome, as well as patients with severe stroke (initial GCS score ≤ 9; male: 6.1%, female: 14.7%) were excluded. Second, the small sample size is another limitation of this study. Based on the values of mtDNA^4977^ in this study, at the level of 0.05, we will need approximate 1,200 case-control pairs to achieve an adequate power of 80% to detect differences in the level of mtDNA^4977^ between the ischemic cases and the control group.

Future work should include a larger and more generalizable cohort to examine correlations between mtDNA^4977^ and clinical stroke occurrence or stroke outcome.

## Conclusions

MtDNA^4977^ does not play a significant role in cerebral infarction. Inability to detect mtDNA^4977^ may be a marker or risk factor for ischemic stroke.
